# Content and Frequency of Writing on Diabetes Bulletin Boards: Does Race Make a Difference?

**DOI:** 10.2196/jmir.1153

**Published:** 2009-06-24

**Authors:** Siobhan Case, Valarie Jernigan, Audra Gardner, Philip Ritter, Catherine A Heaney, Kate R Lorig

**Affiliations:** ^1^Stanford Patient Education Research CenterStanford UniversityPalo AltoCAUSA

**Keywords:** Self-management, race, Internet, patient education, diabetes

## Abstract

**Background:**

Diabetes-related disparities are well documented among racial minority groups in the United States. Online programs hold great potential for reducing these disparities. However, little is known about how people of different races utilize and communicate in such groups. This type of research is necessary to ensure that online programs respond to the needs of diverse populations.

**Objective:**

This exploratory study investigated message frequency and content on bulletin boards by race in the Internet Diabetes Self-Management Program (IDSMP). Two questions were asked: (1) Do participants of different races utilize bulletin boards with different frequency? (2) Do message, content, and communication style differ by race? If so, how?

**Methods:**

Subjects were drawn by purposeful sampling from participants in an ongoing study of the effectiveness of the IDSMP. All subjects had completed a 6-week intervention that included the opportunity to use four diabetes-specific bulletin boards. The sample (N = 45) consisted of three groups of 15 participants, each who self-identified as American Indian or Alaskan Native (AI/AN), African American (AA), or Caucasian, and was stratified by gender, age, and education. Utilization was assessed by counting the number of messages per participant and the range of days of participation. Messages were coded blindly for message type, content, and communication style. Data were analyzed using descriptive and nonparametric statistics.

**Results:**

In assessing board utilization, AAs wrote fewer overall messages (*P* = .02) and AIs/ANs wrote fewer action planning posts (*P* = .05) compared with Caucasians. AIs/ANs logged in to the program for a shorter time period than Caucasians (*P* = .04). For message content, there were no statistical (*P* ≤ .05) differences among groups in message type. No differences were found in message content between AAs and Caucasians, but AIs/ANs differed in content from both other groups. Caucasians wrote more on food behaviors than AIs/ANs (*P* = .01), and AIs/ANs wrote more about physical activity than Caucasians (*P* = .05) and about walking than the other two groups (*P =* .01). There were no differences in communication style.

**Conclusions:**

Although Caucasians utilized the boards more than the other two groups, there were few differences in message type, content, or style. Since participation in bulletin boards is largely blind to race, age, gender, and other characteristics, it is not clear if finding few differences was due to this optional anonymity or because non-Caucasian participants assumed that they were communicating with Caucasians. If the low variability between racial groups indicates that the IDSMP is flexible enough to meet the needs of multiple racial groups, then online programs may be an accessible and effective tool to reduce health disparities. These questions need to be investigated in future studies.

**Trial Registration:**

Parent trials: Clinicaltrials.gov NCT00372463 and NCT00185601; http://clinicaltrials.gov/ct2/show/NCT00372463 and http://clinicaltrials.gov/ct2/show/NCT00185601 (archived by WebCite at http://www.webcitation.org/5hm2g0AeX and http://www.webcitation.org/5hm2i4XVw)

## Introduction

The prevalence of diabetes is growing, and more so in some racial groups than others. American Indians/Alaskan Natives (AIs/ANs) are 2.2 times more likely, and non-Hispanic blacks are 1.8 times more likely, to have diabetes than non-Hispanic whites [1]. Diabetes-related mortality is significantly higher for both groups [2]. Population-specific interventions that emphasize individual health behaviors are often cited as an important approach to address these types of health inequalities [2-4]. However, while much has been written on health disparities, less is known about the beliefs and actions of different racial groups as they deal with diabetes. This type of information is critical in order to develop and evaluate interventions and ensure that they respond to the needs of vulnerable populations.

In approaching this subject, it is important to note that racial groups are not homogeneous. There may well be as many differences within a group as between groups. This may explain why some of the literature finds specific beliefs among racial groups while other studies find few differences. For instance, Caballero documents general factors that can affect patient adherence and physician–patient relationships, such as individual and social interaction, judgment and beliefs about the disease, nutritional preferences, quality of life, and religion and faith [5]. Multiple focus groups with African Americans (AAs) and AIs/ANs have documented variations within these and other themes for people with diabetes [6-14]. However, at least one study by Cox et al directly comparing diabetes attitudes, behaviors, and perceived knowledge between low-income AAs and Caucasians with type 2 diabetes found no significant differences [15]. Given these mixed findings, it is especially important to further examine utilization of diabetes self-management programs as these factors may affect the participation and participatory style of people of different races.

Online programs are an attractive addition to self-management education based on their accessibility and potential for reducing health disparities [16-18]. Internet-based programs are easily available, thereby eliminating barriers such as geographic location, work schedules, transportation, and physical disability [19,20]. Participants are relatively anonymous since factors like age, race, gender, socioeconomic status, and disability are not immediately apparent [21]. Some hypothesize that this unique environment helps participants share otherwise embarrassing or sensitive comments and feel that their contributions are valued for their true “quality” [21,22]. Furthermore, the Internet is increasingly becoming a way to reach underserved populations as access for previously underrepresented groups increases [23,24]. Half of those with chronic conditions or disabilities use the Internet, and of that population, 86% have looked for health information [25]. While data on AIs/ANs are sparse, the PEW Internet & American Life Project found in 2005 that 57% of AAs and 70% of whites go online, and in 2008, 43% of AAs had broadband access [23,26]. Jackson et al found that 89% of AAs in their study were willing to use an online diabetes program if they received free computers, and various studies on the Comprehensive Health Enhancement Support System were successful with older sample populations, people without computer skills, and racial minorities [17,27-29].

Yet despite the growth in Internet access and its increasing uses in health care, we know little about how different racial groups utilize and participate in Internet groups. Content analyses of online forums have provided important insights into the various uses and utilization of message boards, especially in the provision of social support [30-33], gender differences [34,35], and general content and utilization [36-41]. Research has also suggested that the association between Internet use and social support can differ by race [42]. However, much more research is needed to explore potential differences and similarities.

This study used a subset of subjects from a larger trial designed to evaluate the effectiveness of the Internet Diabetes Self-Management Program (IDSMP). The IDSMP’s predecessor, the Chronic Disease Self-Management Program (CDSMP), has been cited by the Agency for Healthcare Research and Quality as an intervention that could have a significant impact on the health status and health care utilization of racial minorities with diabetes [1]. An online version of the CDSMP has also shown significant improvements in health status that were on par with the community-based program [43].

In the larger trial, participants had to be United States residents over the age of 18 years who spoke English, knew how to read, had basic computer skills, and had Internet access. Exclusion criteria included being pregnant and having undergone cancer treatment. There were no other limitations on comorbidities or HbA_1c_. A total of 760 adults with type 2 diabetes were recruited, largely through links from other websites and user groups. In addition, links were placed in emails to employees working for large public service agencies. To assure a diverse population, recruitment was targeted toward websites and user groups that served specific populations, such as AA churches and AI user groups.

All participants in the sample for this study completed the IDSMP. This program was designed to emulate small group interaction via the Internet, and all portions of the program were asynchronous. Participants were known to each other only by self-chosen screen names. Approximately 25 participants took part in each 6-week workshop. The workshop consisted of weekly education modules, peer-moderated bulletin boards, and an internal post office where participants could communicate one-on-one. The four bulletin boards were titled Action Planning, Problem Solving, Celebrations, and Difficult Emotions. Each workshop generated between 500 and 700 messages. The program was specifically designed to be culturally neutral, with cultural specificity being supplied by the moderators and other group members.

A previous content analysis of the bulletin boards in the IDSMP AI pilot study offered important insights into participants’ experience and needs [36]. This study further investigates these areas for AIs/ANs, AAs, and Caucasians through two main research questions: (1) Do participants of different races utilize bulletin boards differently? (2) Do the type of message, content, and communication style differ by race? If so, how?

## Methods

### Participant Sample

The sample for the present study (N = 45) was drawn from participants in 20 IDSMP workshops. It was constructed to consist of three 15-person groups of AAs, AIs/ANs, and Caucasians. Due to a limited number of AA men, the sample included all four AA males who had completed workshops at the time of sample selection. Another 11 AA females were randomly selected to complete the group of 15. These participants were then matched to AI/AN and Caucasian participants by gender, age, and years of education. The Stanford Institutional Review Board approved this research project.

### Board Utilization

Data included all of the bulletin board messages written by each participant. Utilization was determined by counting the number of posts and responses per participant within each bulletin board as well as the range of days each person logged on (days from first log-on to last log-on). Due to several outliers who wrote many responses, we used a nonparametric analysis. Outliers were found in each of the three racial groups. Wilcoxon signed rank tests were utilized to determine differences between all three pairings of racial groups.

### Message Content

The coding unit was one individual message. Messages where one participant responded to another were labeled as “responses,” and all others were considered “posts.” Coding was blind to demographic characteristics. Codes were generated using a hybrid of inductive and deductive methods and were guided by the main social and cultural factors that Caballero cited as considerations for diabetes education programs for racially diverse groups, as well as Lofland’s six areas of description as translated to an online setting [5,44]. The inductive codes were based on the themes found in the literature, such as specific barriers to care and diabetes beliefs [5-14,37,40,44-47]. Deductive codes were based on Grounded Theory and included all codes that did not fit easily into the inductive themes. The full set of 98 codes was combined into 16 nonexclusive codes during multiple coding passes. These codes were organized into three main coding categories: message type (the purpose of the message, such as asking a question or stating a problem), content (the discussion topics), and communication style (the way people address each other, express themselves, and provide support). These categorizations are related to those in previous content analyses, including the purpose of a message, biomedical and socioemotional content, and social cues [37,40,45]. Code validity was assessed by having two researchers double code and compare data from one randomly selected participant from each of the three racial groups. Researchers initially disagreed on and resolved four codes out of 33 messages with a total of 810 coding references, indicating a low incidence of disagreement.

To analyze messages, the percent of a participant’s messages that were labeled with a specific code were averaged by race to control for variations in the number of messages per participant. These numbers, or “mean percent of messages” for a code, were analyzed with analysis of variance (ANOVA), controlling for race. When the ANOVAs were significant (*P* ≤ .05) or when there were seven or more percentage points between racial groups, we utilized *t* tests for further exploration.

## Results

### Participant Sample

The mean age was 53.7, 52.3, and 50.5 years, respectively, for AIs/ANs, AAs, and Caucasians (range 37 to 61). Average years of education clustered closely at 15.7 for AIs/ANs, 16.1 years for AAs, and 15.9 for Caucasians.

### Board Utilization

Participants wrote a combined total of 1067 messages. There were no significant differences in the number of messages for AIs/ANs and AAs. AAs wrote fewer overall messages than Caucasians (*P =* .02), including fewer problem solving posts (W = −66, *P =* .01) and action planning responses (W = −41, .01 < *P* < .02). Between AIs/ANs and Caucasians, the only significant difference was AIs/ANs posting less on action planning (W = −57, *P* *=* .05). See Table 1 for results.

**Table 1:** Differences in message frequency by race^a,b,c^

Board	Message Type	AIs/ANs vs AAs				AAs vs Caucasians				AIs/ANs vs Caucasians			
		W	N_(s/r)_	*z*	*P*	W	N_(s/r)_	*z*	*P*	W	N_(s/r)_	*z*	*P*
Action Planning	Posts	33	13	1.14	.25	−10	8	N/A	N/A	−**57**	**13**	−**1.97**	**.05**
	Responses	−9	5	N/A	N/A	−**41**	**9**	**N/A**	**.02-.01**^d^	−6	10	−0.28	.78
Celebrations	Posts	7	9	N/A	N/A	−7	10	−0.33	0.74	−22	11	−0.96	.34
	Responses	−4	7	N/A	N/A	−14	8	N/A	N/A	−3	6	N/A	N/A
Emotions	Posts	3	9	N/A	N/A	−18	10	−0.89	.37	−17	11	−0.73	.47
	Responses	−19	9	N/A	N/A	−25	9	N/A	N/A	−1	11	−0.02	.98
Problem Solving	Posts	−20	12	−0.76	.45	−**66**	**12**	−**2.57**	**.01**	−43	15	−1.21	.23
	Responses	2	12	0.06	.95	−37	12	−1.43	.15	−20	13	−0.68	.50
All Boards	Posts	7	14	0.20	.84	−**65**	**14**	−**2.02**	**.04**	−43	15	−1.21	.23
	Responses	−6	13	−0.19	.85	−**71**	**14**	−**2.21**	**.03**	−36	14	−1.11	.27
	Messages	−4	13	−0.12	.90	−**83**	**15**	−**2.34**	**.02**	−26	15	−0.72	.47

^a^ W = sum of signed ranks; N_(s/r)_ = number of signed ranks; N/A = not applicable.

^b^ Bold font indicates significance of *P* ≤ .05.

^c^ All *P* values are 2-tailed.

^d^ *P* value determined through exact sampling distribution for 5 < N_(s/r)_ < 9.

Utilization of the bulletin boards was also measured by the range of days participants logged in to the IDSMP. The maximum number of days from first to last log-in was 42. On average, Caucasians had a significantly longer period of activity, with a median of 42 days, than AIs/ANs, with a median of 40 days (W = −65, *P* = .04). There were no significant differences between AAs and other racial groups. It should be noted that the mean range of activity (30 days) for AIs/ANs was much lower than the median. This is because the activity range for six participants was less than half of the workshop. In contrast, all Caucasian and AA participants were active for at least half of the time.

### Message Content

In the qualitative analysis of message codes, a total of 98 codes within message type, content, and communication style were developed and compared. These collapsed into 16 primary codes, shown in Table 2.

**Table 2:** Primary codes in message type, content, and communication style

	Code	Definition
Message Type	Goal Setting	Mentioning a general or specific goal for oneself, with or without a concrete action attached to it.
	Personal Experience	Relating a personal story or happening.
	Question	Explicit requests to other participants for information or follow-up questions.
	Problem Statement	Describing or stating one’s own diabetes-related physical, mental, social, or emotional problem.
Content	Barriers	Physical or mental barriers that the patient believes interferes with his/her own self-care activities.
	Computer Technology	Specific programs, actions, or characteristics related to computers and the Internet.
	Diet	Dietary behaviors, types of food, food recommendations, and feelings around food.
	Emotions	Explicitly expressing emotion or referencing one’s feelings in a message.
	Medicine	Medical treatment or management of diabetes (eg, health care workers, medications, and alternative or natural treatments).
	Physical Activity	Aerobic or non-aerobic physical exercise, including planning, accomplishments, behaviors, and feelings.
	Physical Symptoms	Experiences relating to the body, such as physical symptoms, blood glucose, chronic illnesses, future complications, and weight.
	Personal Life	Aspects related to the participant’s personal life, such as religion, family, friends, work, and acquaintances with diabetes.
	Self-Management	Self-care activities, including physical activity, diet, medication, general self-management strategies, and other healthy lifestyle practices or behaviors.
Communication Style	Additional Text	Stylizing one’s message text in various ways (eg, symbols or adding non-standard letters, punctuation, and capitalization).
	Identification	Mentioning a personal identifying characteristic (eg, age, gender, job, location, name, race, relationships, or serious illnesses).
	Social Support	Providing appraisal, emotional, informational, or tangible support.

For the sample as a whole, codes were considered prevalent if they appeared in 30% or more of each racial group’s messages. Two message type codes satisfied this condition, with 60% of a participant’s messages relating a personal experience and 43% containing a problem statement. Four content codes also met the criteria, with 56% of an average participant’s messages talking about self-management, 54% conveying emotions, 36% including physical activity, and 33% mentioning barriers.

Differences found by comparing codes by race are summarized in Table 3. There were no significant (*P* ≤ .05) differences in any message type codes. Content codes showed three significant differences: Caucasians wrote more than AIs/ANs on food behaviors, AIs/ANs wrote more than Caucasians on physical activity, and AIs/ANs wrote more on walking than did Caucasians or AAs. There were no significant differences in content codes between AAs and Caucasians. For communication style codes, the two significant differences were that AAs revealed their gender more often than AIs/ANs and that AAs revealed their name more often than both other groups. Again, there were no differences between AAs and Caucasians in communication style. See Table 3 for results.

Finally, participants of all three races wrote that the bulletin boards helped them in their self-care efforts.

**Table 3:** Significant differences in mean percent of messages for all codes by race

	Code	Definition	F^a^	P> F^b^	*P*^c^
Style Content Codes	Diet – Food Behaviors	Self-care for food (eg, planning to eat certain foods, scheduling meals, tracking what one eats).	1.54	0.23	.01 Caucasian > AI/AN
	Physical Activity	Aerobic or nonaerobic physical exercise	2.09	0.14	.05 AI/AN > Caucasian
	Physical Activity – Walking	Walking for physical activity, whether outside or on a treadmill	5.17	0.01	.01 AI/AN > Caucasian .01 AI/AN > AA
Communication Codes	Identification – Name	Revealing one’s name by referring to oneself or signing a message	4.78	0.01	.03 AA > Caucasian .01 AA > AI/AN
	Identification – Gender	Revealing gender directly or though relationships and names	3.53	0.04	.01 AA > AI/AN

^a^ F tests from ANOVA, with two degrees of freedom.

^b^ Probability that the F ratio would be greater by chance if the actual ratio were 1.

^c^ *P* ≤ .05 in *t* test between races.

## Discussion

Somewhat surprisingly, this study suggests that participants of different races use Internet bulletin boards similarly. While there are some differences between racial groups concerning program utilization, the reasons for this are not clear. Overall, these results could indicate that there are more similarities than differences in participation and discussion in online programs despite the documented, population-specific diabetes beliefs among racial groups [6-14]. This possibility would support Cox et al’s study implication that diabetes self-management programs do not necessarily need to be race specific [15].

### Board Utilization

The lower message numbers for AAs and lower activity range for AIs/ANs compared with Caucasians could be related to a number of “upstream” factors, such as Internet and computer access, amount of free time, type of employment, and variations between racial groups documented in the literature, such as barriers to care [5-14]. Perceptions of the IDSMP could influence utilization, too, including the level of comfort online, the acceptability of Internet-based programs, perceived workshop benefits, and relationships with other participants. Finally, message numbers could be related to “lurker” participants, or people who read messages but prefer not to write anything, as documented in previous research [20,48]. These differences should be explored in future studies.

### Message Content

One possible explanation for the absence of differences is that participants, regardless of race, had similar diabetes self-management experiences and issues. The sample characteristics could play a role as well since participants were highly educated, self-selected, and had Internet access. A third possibility is that AAs or AIs/ANs could have thought that they were the only members in their workshops who were racial minorities and therefore did not express themselves the same as they would have in a group of the same race. All of these hypotheses would be interesting directions for future research.

### Limitations

Since it was necessary to draw participants from across workshops, messages were taken out of context, which may have affected interpretation of message content and social support. The blindness of the coding was affected by two AI/AN participants who revealed their race in messages and 26 participants who revealed their gender. Results should be interpreted in light of the participants’ high education level, age, and preexisting Internet access. In addition, the sample was small, included a limited number of males, and was not randomly selected. This makes it difficult to assess the significance of the results and may obscure additional differences between racial groups. A Bonferroni correction was not possible due to the sample size, so the likelihood of false positives is high. While not ideal, these restrictions are necessary tradeoffs given the demographic composition of the overall IDSMP sample and the need to begin researching online program experiences of racial minority groups.

### Conclusion

Bulletin boards are included in the IDSMP based on the hypothesis that sharing experiences and support with other participants can positively impact self-efficacy and potentially improve health outcomes. In order to support participants of all races in the fullest way possible, it is important to explore why AAs and AIs/ANs used message boards less than Caucasians. Additionally, the low variability in messages, outside of sheer numbers, suggests that when participants of different races do use the boards, they use them very similarly, and that there is higher variation within groups than between them. This could indicate that online programs such as the IDSMP are flexible enough to meet the needs of multiple racial groups. If this finding is borne out in further studies, message boards, and by extension, listservs, may be a means of helping to lessen racial disparities by providing readily accessible and effective self-management education.

## Abbreviations

AA: African American
AI: American Indian
AN: Alaskan Native
CDSMP: Chronic Disease Self-Management Program
IDSMP: Internet Diabetes Self-Management Program
